# Observationally-constrained projections of an ice-free Arctic even under a low emission scenario

**DOI:** 10.1038/s41467-023-38511-8

**Published:** 2023-06-06

**Authors:** Yeon-Hee Kim, Seung-Ki Min, Nathan P. Gillett, Dirk Notz, Elizaveta Malinina

**Affiliations:** 1 Division of Environmental Science and Engineering, Pohang University of Science and Technology, Pohang, South Korea; 2 Institute for Convergence Research and Education in Advanced Technology, Yonsei University, Incheon, South Korea; 3 Canadian Centre for Climate Modelling and Analysis, Environment Climate Change Canada, Victoria, BC Canada; 4 Center for Earth System Research and Sustainability (CEN), Institute of Oceanography, Universität Hamburg, Hamburg, Germany

**Keywords:** Projection and prediction, Climate and Earth system modelling

## Abstract

The sixth assessment report of the IPCC assessed that the Arctic is projected to be on average practically ice-free in September near mid-century under intermediate and high greenhouse gas emissions scenarios, though not under low emissions scenarios, based on simulations from the latest generation Coupled Model Intercomparison Project Phase 6 (CMIP6) models. Here we show, using an attribution analysis approach, that a dominant influence of greenhouse gas increases on Arctic sea ice area is detectable in three observational datasets in all months of the year, but is on average underestimated by CMIP6 models. By scaling models’ sea ice response to greenhouse gases to best match the observed trend in an approach validated in an imperfect model test, we project an ice-free Arctic in September under all scenarios considered. These results emphasize the profound impacts of greenhouse gas emissions on the Arctic, and demonstrate the importance of planning for and adapting to a seasonally ice-free Arctic in the near future.

## Abstract (web summary)

A dominant influence of anthropogenic greenhouse gas increases on Arctic sea ice area is detectable in all months. By scaling climate models’ sea ice response to best match observed trends, an ice-free Arctic in September is projected under all scenarios.

## Introduction

Arctic sea ice area (SIA) has been declining rapidly throughout the year during recent decades with a steeper decline since 2000. Based on a model selection approach applied to Coupled Model Intercomparison Project phase 6 (CMIP6) models, Notz et al.^1^ projected that the Arctic Ocean will become sea ice-free in September for the first time before 2050, irrespective of emission scenarios. However, the Sixth Assessment Report of the Intergovernmental Panel on Climate Change^2^ assessed that “it is *likely* that the Arctic Ocean in September, the month of annual minimum sea ice area, will become practically ice-free (SIA < 1 × 10^6^ km^2^) averaged over 2081–2100 and all available simulations” only under the SSP2-4.5, SSP3-7.0, and SSP5-8.5 scenarios. Hence, uncertainty remains in whether or not the Arctic will become ice-free under the lowest emissions scenarios, as well as in understanding the observed Arctic SIA changes throughout the year.

Previous optimal detection and attribution studies have quantified the net human contribution to the observed Arctic sea ice melting^3–5^. By comparing Arctic sea ice extent observations during 1953–2006 with CMIP3 multi-model simulations, anthropogenic influences on the observed reduction were found to be detectable from the early 1990s onwards^3^. The study also detected the anthropogenic signals for individual months from May to December. Another study^4^ compared the observed September Arctic sea ice extent from 1979 to 2012 with that from CMIP5 models and two single-model ensembles and detected the anthropogenic signal separately from the natural forcing (solar and volcanic) signal, which was confirmed later using four observational data sets^6^. On the other hand, the cooling contribution of anthropogenic aerosols to the observed annual Arctic sea ice extent increase from the 1950s to the 1970s was identified using CanESM2 simulations^7^. A recent study^5^ further detected the influences of natural, greenhouse gas (GHG), and other anthropogenic (mainly anthropogenic aerosol) forcing on the observed September Arctic sea ice extent change during 1953–2012 using eight CMIP5 models and showed that anthropogenic aerosol forcing has offset about 23% of the GHG-induced Arctic sea ice extent decrease.

CMIP5 models underestimate significantly the observed trend in Arctic sea ice decline^8^, inducing uncertainty in future sea ice projections. In view of this, methods of constraining projections with observations have been applied^9–11^. Those methods were based on the statistical relationship between sea ice cover trends in the past and future^12^, between global mean temperature and SIA^9^, between the current seasonal cycle and future change in sea ice albedo feedback^10^, or between historical model performance and projected future Arctic sea ice extent^11^. Scaling factors (i.e., regression coefficients) between modeled responses and observations derived from optimal detection studies can provide a more rigorous way to constrain climate projections^13–17^ when combined with an assessment of the validity of constrained projections using an imperfect model test^18, 19^.

The present study conducts an updated detection and attribution analysis of the observed Arctic SIA changes across all months over the 1979–2019 period by comparing three satellite observations with the CMIP6 multi-model simulations. Previous attribution studies were mostly based on sea ice extent which is defined as the total area of all grid cells with at least 15% sea ice concentration and hence is strongly grid dependent^1^. Here we use SIA which is defined as the actual area covered with sea ice and is more appropriate for comparison with satellite observations than sea ice extent^20^, having a smaller observed uncertainty than sea ice extent^21^. The Arctic sea ice has been melting in all months^22–24^ but most previous studies have focused on September when the largest change occurred. Considering all calendar months and utilizing individual forcing simulations, this study finds that the response to GHG increases is detected throughout the year, explaining most of the observed SIA reduction. Further, based on the quantified GHG contribution to the observed Arctic SIA reduction, the future timing of the ice-free Arctic Ocean is projected under the different Shared Socioeconomic Pathway (SSP) emission scenarios.

## Results

### Observed and modeled SIA changes

**Fig. 1:**
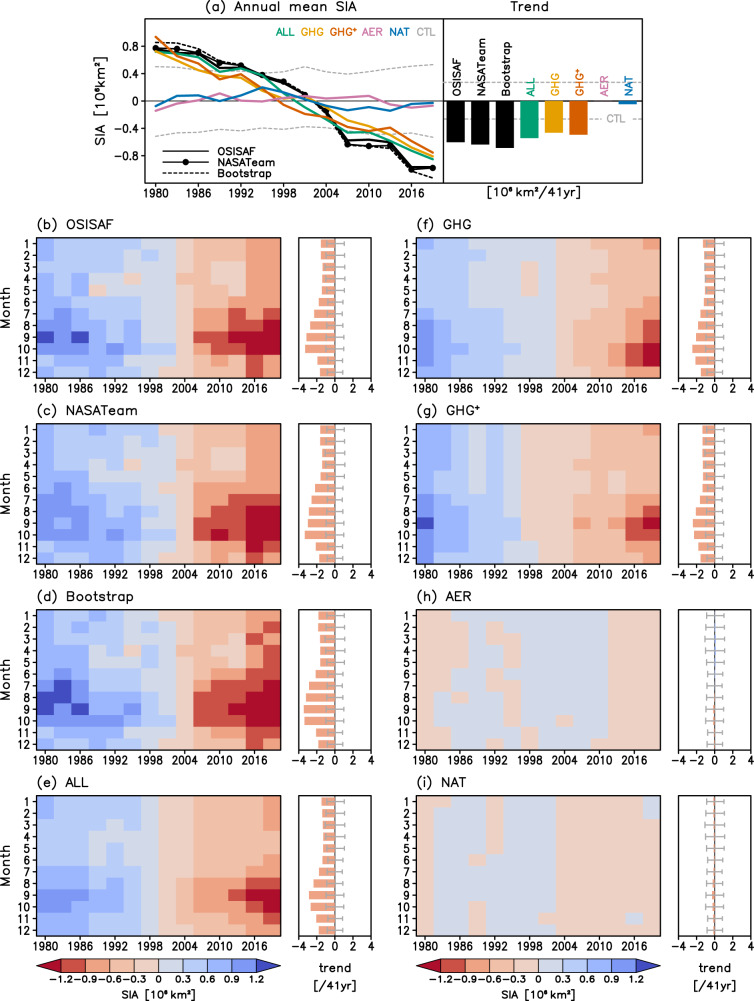
Time series of observed and simulated Arctic sea ice area (SIA) and their linear trends. 3-year mean time series of observed and simulated Arctic SIA for 1979–2019 for **a** annual mean (line plot) and **b**–**i** each calendar month (shading), and their linear trends (bars) and 5–95% ranges (gray dashed lines or error bars) estimated from the preindustrial control (CTL) simulations. Three observations (OBS) from OSISAF, NASATeam, and Bootstrap are compared with Coupled Model Intercomparison Project Phase 6 (CMIP6) multi-model simulations from historical (ALL; anthropogenic plus natural forcing), hist-GHG (GHG; well-mixed greenhouse gas only forcing), hist-aer (AER; anthropogenic aerosol-only forcing) and hist-nat (NAT; natural only forcing) experiments. The residual GHG distribution (GHG^+^) obtained by All–AER–NAT is displayed. SIA anomalies are obtained by computing non-overlapping 3-year averages (2-year averages for 2018–2019) relative to the 1979–2019 means. ALL runs are extended by using Shared Socioeconomic Pathway (SSP) 2–4.5 scenario runs since 2015.

Observed and model-simulated Arctic SIA changes are compared first. Figure 1 displays the anomaly time series of 3-year mean Arctic SIA and their linear trends during the past 41 years (1979–2019) for all calendar months from three observational data sets and CMIP6 multi-model means (MMM) for anthropogenic plus-natural (ALL), greenhouse gas only (GHG), aerosol only (AER), and natural only (NAT) forcings (see Methods). Annual mean results are provided at the top for comparison and the 5–95th percentile values of preindustrial control (CTL) runs (gray dashed lines) are added to measure the internal variability ranges. Three sets of observations (OSISAF, NASATeam, and Bootstrap; see Methods) consistently exhibit decreasing trends throughout all calendar months, with stronger amplitudes in warm seasons than in cold seasons, consistent with previous studies^1^. Bootstrap data show slightly stronger trends while OSISAF data have relatively weak trends as can be seen in annual mean trends. These observed trends are beyond the internal variability ranges for all calendar months, even in cold months, confirming the significant year-round melting of Arctic sea ice during recent decades.

The temporal evolution patterns of ALL simulations are overall consistent with those observed, capturing year-round significant melting and the strongest melting during September–October. However, decreasing trends in ALL are on average weaker than observations, particularly in the warm season (also see Fig. S2). GHG runs also resemble the observed melting patterns but exhibit slightly weaker trends than ALL runs, suggesting other forcing influences like tropospheric ozone or reductions in aerosol emissions may slightly increase the trends in ALL. Note that while previous studies indicate an aerosol-driven increase in SIA since 1950, CMIP5 simulations, consistent with the simulations shown here, show little change in SIA in response to aerosols in the period since 1980^5^. Indeed, GHG^+^ patterns (constructed as ALL–AER–NAT, see Methods), which reflect the combined response to increases in all greenhouse gases including tropospheric ozone^19^, show stronger sea ice melting than GHG patterns (which reflect the response to well-mixed greenhouse gases only) in annual time series, long-term trends, and seasonal evolutions. NAT forcing shows a slight decrease in Arctic SIA for all calendar months, contributing to the observed trend. This NAT-induced sea ice melting seems to be associated with no major volcanic eruptions after Pinatubo (1991), as can be clearly seen from the annual mean time series. Actually, Arctic SIA in NAT has a maximum near 1995, consistent with the previous finding^6^ that Arctic sea ice extent peaks 5 years after volcanic eruptions. A small increase in Arctic SIA after El Chichón eruption (1982) is noticeable as well. AER runs exhibit negligible long-term trends in Arctic SIA over the 1979–2019 period with a seasonal contrast—increasing in winter and spring and decreasing in summer and fall. The negative Arctic SIA trend in the warm season might be partly related to the observed decreases in anthropogenic aerosol emissions since the 1980s^7^.

### Attribution results

**Fig. 2:**
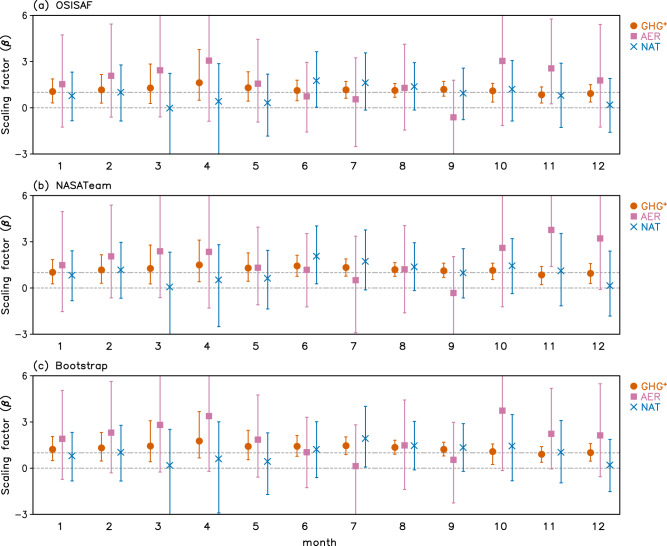
Results of three-signal (GHG+, NAT, and AER) detection. Three-way regression (GHG^+^, NAT, and AER) results for the observed changes in Arctic sea ice area (SIA) for each month for three observational data sets of **a** OSISAF, **b** NASATeam, and **c** Bootstrap. The best estimates (marks) and 5–95% ranges (error bars) of scaling factors (regression coefficients, see Methods) are displayed for each signal. Using 3-year mean anomaly time series of Arctic SIA (see Fig. 1), observations are regressed simultaneously onto multi-model-simulated responses (fingerprints) to three signals of GHG^+^, NAT, and AER forcing (see Methods).

Three-signal detection analyses are conducted by regressing the observations onto GHG^+^, AER, and NAT (i.e., in a three-way regression, see Methods). Figure 2 shows results for each calendar month obtained using OSISAF, NASATeam, and Bootstrap observations. GHG^+^ signals are detected in all calendar months from all observations while almost no detection occurs for AER and NAT signals. This result indicates a GHG^+^ influence on every month’s Arctic SIA decrease, which is separable from AER and NAT signals. In most of the detected cases, the 90% range of scaling factors (regression coefficients, see Methods) includes unity, indicating consistency in amplitude with the observed changes. However, best estimates of the GHG^+^ scaling factor are generally larger than unity except for November-December, meaning that models on average underestimate the observed Arctic SIA decrease over the past 41 years, as shown above in Fig. 1. Consequentially, these results clearly show that GHGs have contributed considerably to the observed Arctic SIA decrease for all calendar months with some underestimation by models.

**Fig. 3:**
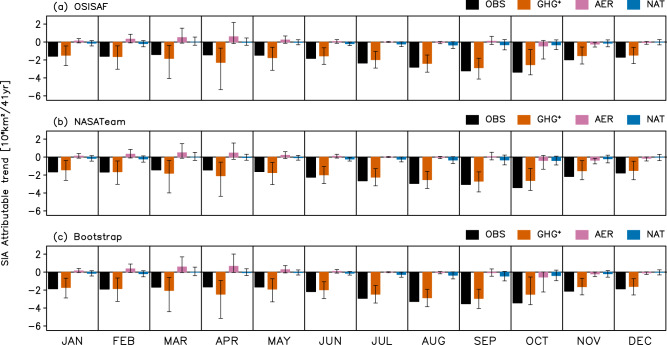
Attributable trends. Attributable trends (color bars) in Arctic sea ice are (SIA) for each month during 1979–2019 to GHG^+^, AER, and NAT forcings compared with the observed trends (OBS, black bars) from **a** OSISAF, **b** NASATeam, and **c** Bootstrap. Attributable trends (see Methods) are obtained based on scaling factors from the three-signal analysis shown in Fig. 2 by multiplying each signal’s fingerprint (3-year mean SIA anomaly time series) by the corresponding scaling factors (best estimates). The error bars indicate the 5–95% intervals of attributable trends calculated using the 5–95% ranges of scaling factors.

To quantify the relative contribution of the three forcings to the observed Arctic SIA changes, attributable trends are estimated from scaled fingerprints (3-year mean SIA time series multiplied by regression coefficients) for GHG^+^, AER, and NAT (Fig. 3, See Methods). Results show that GHG^+^ explains most of the observed Arctic SIA decline across all calendar months and based on all observation data sets. The NAT contribution to the Arctic SIA decrease is up to about 10% from July to November while AER exerts a negligible influence except for causing a slight increase from February to May. The limited contribution of anthropogenic aerosols to the observed Arctic SIA change is in line with no overall long-term trend in AER time series during the analysis period (Fig. 1). This might be due to the reduced aerosol emissions over Europe and North America since the 1980s, which could offset the cooling effect of increased aerosol emissions in Asia^25–28^. Overall, GHG increases are found to be the main driver of the observed Arctic SIA reduction throughout the year.

Some previous studies have emphasized the role of internal variability in driving SIA trends^29, 30^, and have argued, based on observations of atmospheric circulation change, that internal variability has intensified sea ice decreases over recent decades^29^. Other studies suggest that climate models underestimate multi-decadal internal variability in extratropical temperature and precipitation^31, 32^. While we restrict our attention to observations and simulations of sea ice extent itself, our optimal fingerprint analysis accounts for the influence of internal climate variability by using simulated internal variability from the preindustrial control simulations to estimate uncertainties in regression coefficients. This approach is in turn validated by checking the consistency of observed and model-simulated variability using a residual consistency test (see Methods). Results show that SIA for all months passes the residual consistency test (Fig. 2, no marks under scaling factors), meaning that the modeled variability is consistent with the observed residual variability. Further, based on a comparison of power spectra (Fig. S3), on inter-annual to decadal time scales, the CMIP6 multi-model mean exhibits similar variability of SIA to the observations, and the observed power spectra lie within the inter-model range. This indicates that the models used in this study do not underestimate SIA variability, and hence our estimates of attributable trends and their uncertainties are expected to be reliable.

### Observationally-constrained future SIA

**Fig. 4:**
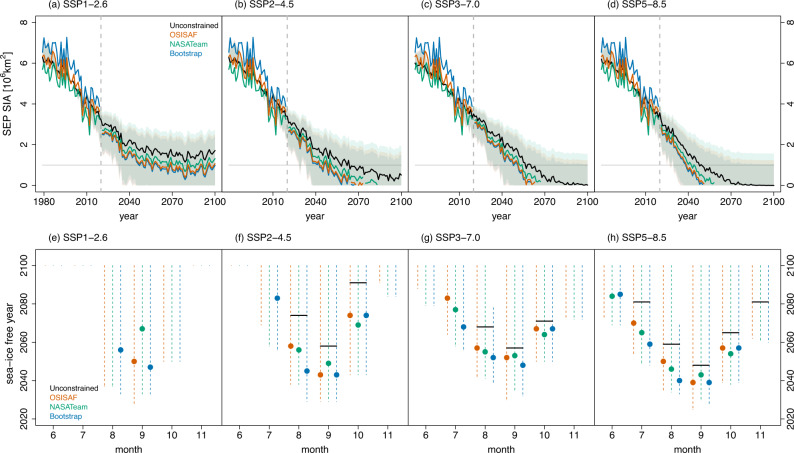
Observationally-constrained future projection. Time series of Arctic sea ice area (SIA) in September over the period 1979–2100 from three sets of observations (OSISAF, NASATeam, and Bootstrap) and Coupled Model Intercomparison Project Phase 6 (CMIP6) multi-model means of the **a** Shared Socioeconomic Pathway (SSP) 1–2.6, **b** SSP2-4.5, **c** SSP3-7.0, and **d** SSP5-8.5 scenario simulations. Colored lines indicate observations for the historical period (1979–2019) and observationally-constrained projections for the future period (2020–2100) which are obtained by scaling raw projections (black lines) with GHG scaling factors (see Fig. 2). Colored shading indicates the uncertainty ranges (5–95%) of observationally-constrained projections (based on 5–95% ranges of GHG^+^ scaling factors). **e**–**h** CMIP6 projected sea ice-free years (defined as the year when monthly mean multi-model mean SIA drops below 1 × 10^6^ km^2^ for the first time) for different SSP scenarios. Marks and vertical dashed lines indicate the best estimate and 5–95% ranges, respectively, of constrained projection results based on GHG scaling factors for three observations. Horizontal lines indicate results from unconstrained projections.

Using the scaling factors for the detected GHG^+^ signal, and their associated uncertainty intervals, observationally-constrained future projections of Arctic SIA are obtained and sea ice-free years are estimated based on multi-model means for four SSP scenarios (SSP1-2.6, SSP2-4.5, SSP3-7.0, and SSP5-8.5). This is done for the three observational data sets separately, to assess the influence of observational uncertainty. Figure 4a–d show results for September Arctic SIA. Unconstrained raw projections (black lines) indicate decreases in Arctic SIA with different slopes depending on SSP scenarios. Except for SSP1-2.6, Arctic SIA decreases past the ice-free threshold (1 × 10^6^ km^2^) around the 2050s–2060s in the unconstrained projections, but it does not reach this threshold in the SSP1-2.6 scenario (also see Fig. 4e, f). This is consistent with the full-model ensemble based projections^1, 23^.

The observationally-constrained projections (colored lines) exhibit a faster decline of Arctic SIA than the raw time series, which can be seen in all SSP scenarios (Fig. 4a–d). This faster retreat of Arctic sea ice in constrained projections is due to GHG^+^ scaling factors larger than unity, which reflects CMIP6 models’ underestimation of the observed trend as discussed above. The stronger SIA declines have advanced first ice-free years to around the 2030s–50s in all SSP scenarios. Importantly, the earlier occurrence of an ice-free Arctic in September becomes evident even in the SSP1-2.6 low emission scenario in all observations (circle marks in Fig. 4e). It is also found that an ice-free Arctic before 2100 occurs in other months (Fig. 4e–h). There are clear differences between scenarios, e.g., with an ice-free Arctic in August to September in SSP2-4.5 vs. June to October in SSP5-8.5, representing more ice-free months under a higher emission scenario. Overall, our constrained projections indicate a faster arrival of an ice-free Arctic by around a decade, than previous studies based on CMIP5 models^10, 33^.

To evaluate the constrained projection approach, we conducted an imperfect model test using pseudo observations of SIA (see Methods). Results show that the correlation coefficients between constrained SIA and simulated SIA for 2031–2050 ranges from 0.44 to 0.57 across four SSP scenarios, all of which are statistically significant (Fig. S4). Also, root mean square errors (RMSEs) from constrained projections are reduced (0.71–0.88 × 10^6^ km^2^) compared to the unconstrained cases (0.95–1.39 × 10^6^ km^2^). These high correlations and reduction in RMSE indicate good predictability for GHG-constrained SIA irrespective of future emission scenarios.

## Discussion

This study conducts an attribution analysis of the observed Arctic SIA decrease for all calendar months for the past 41 years (1979–2019). We compare Arctic SIA from three observational data sets with those from CMIP6 multi-model simulations under different external forcings using an optimal fingerprinting technique. All three observational data sets show a significant Arctic SIA reduction for all calendar months, which is beyond the range of internal variability ranges, with the strongest trends in the summer months. The ALL and GHG simulations successfully reproduce the observed seasonal pattern of trends in Arctic SIA although magnitudes are underestimated, particularly in the warm season. The NAT simulations exhibit slight decreasing Arctic SIAs due to no major volcanic eruptions after Pinatubo (1991), whereas the AER runs show almost no trends in Arctic SIA with a contrasting seasonal pattern.

An optimal detection analysis based on a three-way regression is conducted by regressing observations onto the ALL, AER, and NAT fingerprints simultaneously. Results show that GHG^+^ influences (estimated as ALL–AER–NAT) are detected separately from the responses to AER and NAT forcings for all calendar months from all observations. An analysis of attributable changes indicates that most of the observed Arctic SIA reduction is explained by GHG^+^ forcing with much weaker contributions from AER and NAT. Based on the GHG^+^ scaling factors, we produce observationally-constrained future changes in Arctic SIA under four SSP scenarios. Results indicate that the first sea ice-free September will occur as early as the 2030s–2050s irrespective of emission scenarios. Extended occurrences of an ice-free Arctic in the early summer months are projected later in the century under higher emissions scenarios.

This study demonstrates that GHG forcing has dominated the observed Arctic SIA reduction across all months, and shows that the GHG influence is separable from that of other factors including anthropogenic aerosols, solar and volcanic forcing, as well as natural internal variability. This result builds on previous CMIP3 and CMIP5-based findings^3–5^ and demonstrates an expanded human influence on the Arctic cryosphere. Our observationally-constrained projections based on attribution results also suggest that we may experience an unprecedented ice-free Arctic climate in the next decade or two, irrespective of emission scenarios. This would affect human society and the ecosystem both within and outside the Arctic, through changing Arctic marine activities^34^ as well as further accelerating the Arctic warming and thereby altering Arctic carbon cycling^35, 36^.

## Methods

### Observations

As observations, we use three different satellite data sets of sea ice concentration (SIC) derived from OSISAF^37^, NASATeam^38^, and Bootstrap^39^ algorithms for each month from 1979 to 2019. Arctic SIA is calculated as the area sum of grid cells weighted by SIC over the Northern Hemisphere (NH).

### CMIP6 simulations

We use multi-model CMIP6 historical and DAMIP simulations^40, 41^ performed under different climate forcing combinations, including historical (anthropogenic plus natural forcing, referred to as ALL), hist-GHG (well-mixed greenhouse gas only forcing; GHG), hist-aer (anthropogenic aerosol-only forcing; AER) and hist-nat (natural only forcing; NAT) for the 41-year period 1979 to 2019. For ALL, historical simulations for 1979–2014 are concatenated with the corresponding Shared Socioeconomic Pathway (SSP)^42^ 2–4.5 scenario simulations for 2015–2019. We also use preindustrial control simulations (CTL) from 38 models which provide 516 41-year non-overlapping chunks for estimating internal climate variability (see below). For future projections of Arctic SIA, we use four SSP scenario simulations (SSP1-2.6, SSP2-4.5, SSP3-7.0, and SSP5-8.5) from the same models for 2020–2100. Following ref. ^1^, modeled Arctic SIA is obtained by multiplying SIC on the ocean grid with the individual grid-cell area and taking its sum over the NH.

We use 10 CMIP6 models which provide all experiments including ALL, GHG, AER, and NAT over the 1979–2019 period. These models can simulate SIA seasonal cycle similar to the observed (Fig. S1). To reduce possible influences of different models, we use the same multi-model ensemble to estimate ‘fingerprints’, i.e., model’s response patterns to external forcings (10 models and 60 runs, Table S1). We use an equal number of ensemble members for each forcing for each model. When comparing the multi-model mean (MMM) from the 10 selected models with that from the 25 models, SIA seasonal cycle and its trends in September and March are found to be very similar (Figs. S1 and S2), indicating that the selected models represent full available models reasonably well. Individual model means are first calculated using all ensemble members and then the MMM is obtained by taking averages of individual model means. In order to reduce noise on inter-annual time scales, we use 3-year mean (2-year mean for 2018–2019) non-overlapping time series of Arctic SIA during 1979–2019 for each month.

### Optimal fingerprinting analysis

To compare the observed Arctic SIA changes with those from CMIP6 forced simulations, we employ the total least squares (TLS)-based optimal fingerprinting method^43^, referred to as regularized optimal fingerprinting (ROF^44^), which provides an improved estimate of the covariance matrix of internal variability. We carry out a three-signal analysis (i.e., three-way regression) to detect the influence of each signal separately. Observations (OBS) are regressed onto ALL, AER and NAT fingerprints simultaneously: OBS = *β*_1_X_ALL_ + *β*_2_X_AER_ + *β*_3_X_NAT_ + ε. The fingerprint X is obtained as 3-year mean time series of the multi-model mean SIA anomaly for the period 1979–2019 for each external forcing (ALL, AER and NAT). Here *β* is the scaling factor (or regression coefficient) for a given external forcing and estimated based on the TLS method. ε represents the internal climate variability estimated from CTL simulations. Scaling factors for GHG^+^, AER and NAT are obtained by decomposing ALL as ALL = GHG^+^ + AER + NAT, where GHG^+^ includes land use change, as well as ozone and well-mixed GHG changes, and substituting as follows: OBS = *β*_1_X_GHG_^+^ + (*β*_1_ + *β*_2_)X_AER_ + (*β*_1_ + *β*_3_)X_NAT_ + ε. The scaling factors for GHG^+^, AER and NAT can hence be written as *β*_GHG_^+^ = *β*_1_, *β*_AER_ = *β*_1_ + *β*_2_, *β*_NAT_ = *β*_1_ + *β*_3_. Two sets of CTL simulations are used (with 258 CTL segments in each, Table S1). The first set is used to estimate covariance matrix of internal variability and hence the best estimate of *β*, and the second set is utilized to estimate confidence interval (5–95%) and also to carry out a residual consistency test^44^. The observed residual (OBS_res_) is obtained by removing the forcing-explained portion from observations based on the optimal regression, i.e., OBS_res_ = OBS – (*β*_1_X_ALL_ + *β*_2_X_AER_ + *β*_3_X_NAT_). Then the variance in residual observations is compared with that in the unforced CTL simulations, using an *F*-test. When the test is failed due to too small modeled variability, detection results become less robust.

If confidence intervals on *β* lie above zero, this indicates signal ‘detection’ (i.e., the observed change is influenced by external forcing). If confidence intervals on *β* include unity, an ‘attribution’ statement can be made that the observed changes are consistent with the simulated response to the external forcing^45^. Attributable trends in Arctic SIA to each forcing are estimated by multiplying GHG^+^, AER and NAT fingerprints by the corresponding scaling factors obtained from the three-signal analysis, and their associated uncertainties.

### Observationally-constrained projections

These scaling factors are further used to produce observationally-constrained future projections of Arctic SIA for four SSP scenarios (SSP1-2.6, SSP2-4.5, SSP3-7.0, and SSP5-8.5). We first obtain multi-model mean time series of SIA anomalies during 2020–2100 relative to the 1979–2019 mean of multi-model mean ALL. The multi-model mean projections of SIA anomalies are weighted by scaling factors of GHG^+^ (based on three-signal analysis). Then, the 1979–2019 multi-model mean climatology is added to the constrained SIA anomaly time series to get the constrained projections of SIA. Finally, sea ice-free year is defined in the raw and constrained projections as the first year when Arctic SIA becomes less than 1 × 10^6^ km^2^ following ref. ^1^. To evaluate this constraining approach, we adapt an imperfect model framework^18, 19^. We treat a single ensemble member from the set of ALL simulations (60 runs) as pseudo observations and use the other 9 models to estimate multi-model mean fingerprints (for GHG^+^, AER, and NAT) and carry out an optimal fingerprinting analysis using the pseudo observations. If the GHG^+^ signal is detected, we constrain the future projections from the 9 models using the scaling factor of GHG^+^. Finally, we compare the constrained SIA with the future simulated SIA obtained from the pseudo-observation ensemble for 2031–2050 when most models simulate ice-free years in September. This procedure is repeated 60 times using each member of ALL simulations.

## Supplementary information

Supplementary Information

## Data Availability

All the raw CMIP6 model simulation data are publicly available at https://esgf-node.llnl.gov/projects/cmip6/. The observed SIA is available at 10.25592/uhhfdm.8559.

## Code availability

The code for optimal fingerprinting analysis is available at https://github.com/ESMValGroup/ESMValTool/blob/gillett20/esmvaltool/diag_scripts/attribute/.
