# Supplementary material for: Observationally-constrained projections of an ice-free Arctic even under a low emission scenario
Source: Nat Commun. 2023 Jun 6;14:3139. doi: 10.1038/s41467-023-38511-8 (PMC10244426; doi:10.1038/s41467-023-38511-8)
Supplement: Supplementary file 1 — Supplementary Information [file 41467_2023_38511_MOESM1_ESM.pdf]

Supplementary Information for

# Observationally-constrained projections of an ice-free Arctic even under a low emission scenario


Yeon-Hee Kim[1], Seung-Ki Min[1,2*†], Nathan P. Gillett[3], Dirk Notz[4], Elizaveta Malinina[3]

[1]*Division of Environmental Science and Engineering, Pohang University of Science and Technology, Pohang, South Korea*

[2]*Institute for Convergence Research and Education in Advanced Technology, Yonsei University, Incheon, South Korea*

[3]*Canadian Centre for Climate Modelling and Analysis, Environment Climate Change Canada, Victoria, BC, Canada*

[4]*Center for Earth System Research and Sustainability (CEN), Institute of Oceanography, Universität Hamburg, Hamburg, Germany*

[*] Corresponding author:
[†] E-mail: skmin@postech.ac.kr

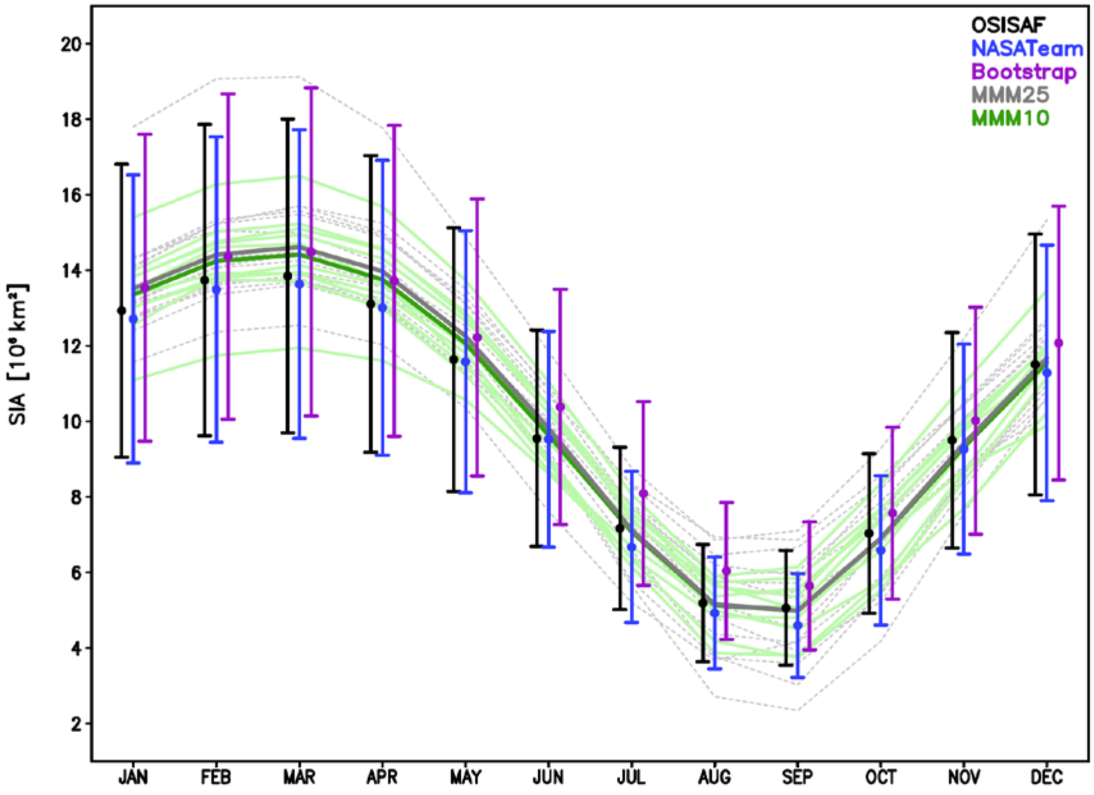


**Fig. S1 | Seasonal cycle of Arctic sea ice area (SIA).** Seasonal cycle of Arctic SIA from three sets of observations (OSISAF, NASATeam, and Bootstrap) and historical simulations (ALL) from 25 Coupled Model Intercomparison Project Phase 6 (CMIP6) models and their multi-model means (MMM) over 1979-2019. Thin and thick green solid lines indicate 10 individual models (used in this study; see Table S1) and their MMM, respectively. Dotted gray lines show the other 15 models and thick grey line indicates the MMM from 25 models. The error bars indicate ±30% range of the observed SIA for each month.

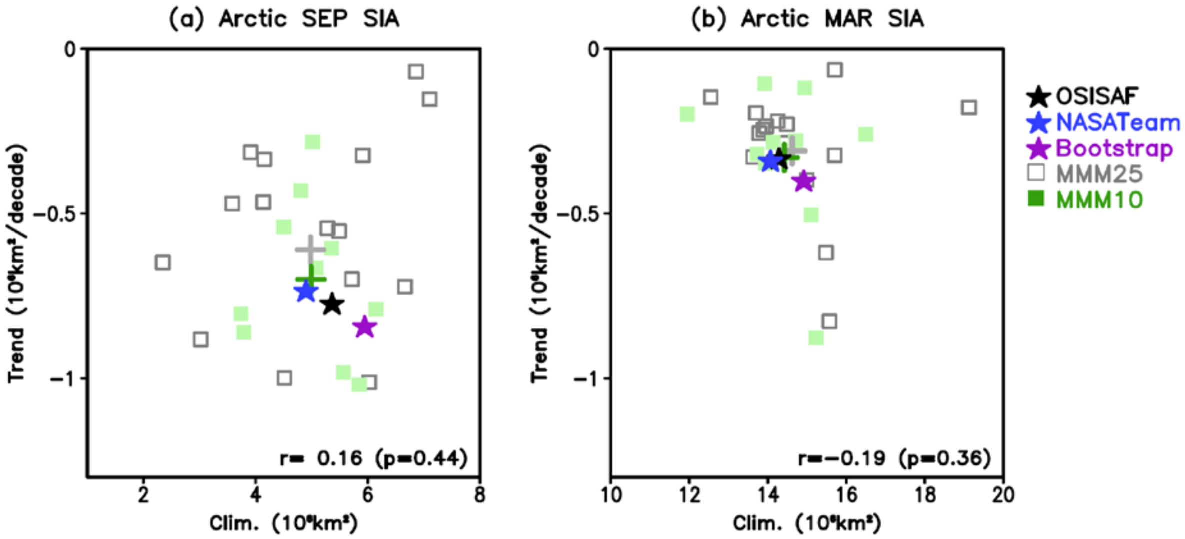


**Fig. S2 | Scatter plot of climatology and trend**. Climatology (x-axis) and trend (y-axis) in Arctic sea ice area (SIA) in (a) September and (b) March for 1979-2019 from three observations and Coupled Model Intercomparison Project Phase 6 (CMIP6) historical simulations (ALL). Green square and cross indicate 10 models and their multi-model means (MMM10) while grey marks represent same for 25 models (MMM25; see Table S1).

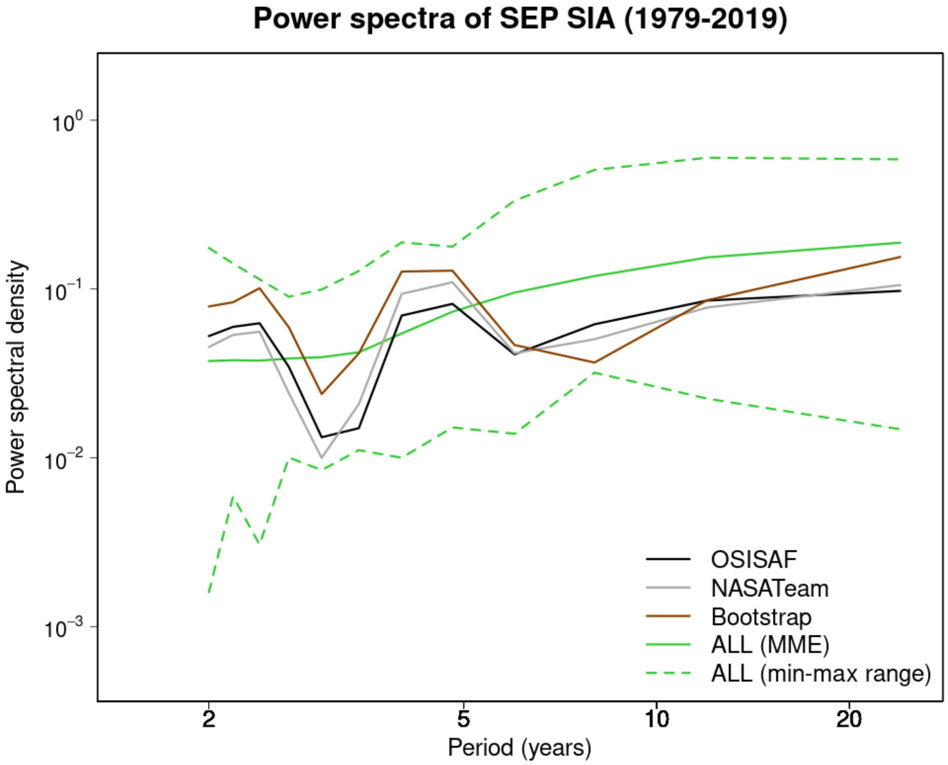


**Fig. S3 | Power spectra of September Arctic sea ice area.** Results from three observations (OSISAF, NASATeam, and Bootstrap) are compared with those from Coupled Model Intercomparison Project Phase 6 (CMIP6) historical simulations (ALL) for 1979-2019. The green solid line indicates the multi-model mean of the power spectral density and green dashed lines represent the min-max range from individual models.

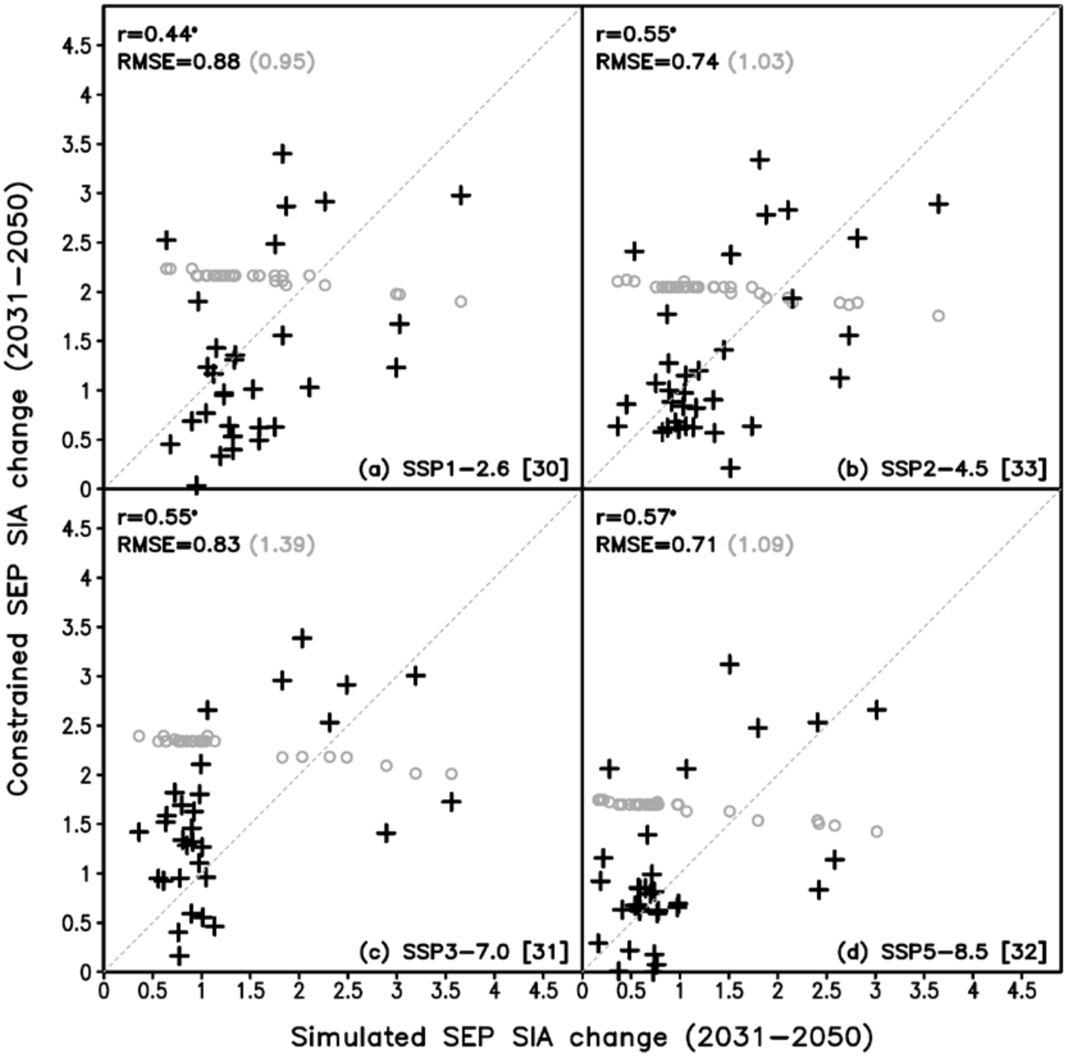


**Fig. S4 | Imperfect model test of attributable Arctic sea ice area (SIA).** Scatter plots between simulated SIA (x-axis) and constrained SIA (y-axis) for 2031-2050 for four Shared Socioeconomic Pathway (SSP) scenario simulations (units: $10^6$ km$^2$). Correlation coefficients (r) between simulated and constrained SIA are shown, with statistical significance at the 5% level indicated by asterisks. Root mean square error (RMSE) values obtained from constrained projections (black plus marks) are compared with those from unconstrained projections (grey circle marks). Simulated SIA is obtained from the raw future simulation [corresponding to the ensemble member of Coupled Model Intercomparison Project Phase 6 (CMIP6) historical simulations (ALL)] used as pseudo-observations. Unconstrained SIA is calculated from multi-model means of 9 models (excluding the model providing the presudo-observation member). Constrained SIA is calculated from constrained multi-model means [i.e. weighted with the scaling factor of corresponding residual GHG forcing (GHG$^+$, see Methods)]. Numbers in square brackets represent total number of cases out of 60 peudo-observations with GHG$^+$ signal detected.

**Table S1.** List of Coupled Model Intercomparison Project Phase 6 (CMIP6) model simulations used in this study. Numbers represent the ensemble size for historical (anthropogenic plus natural forcing; ALL), hist-nat (natural only forcing; NAT), hist-GHG (well-mixed greenhouse gas only forcing; GHG), hist-aer (anthropogenic aerosol only forcing; AER) and four Shared Socioeconomic Pathway (SSP) experiments and non-overlapping 41-year chunks for preindustrial control (CTL) simulations. Models with asterisks represent 10 models used to estimate fingerprints, which provide data for ALL, NAT, GHG and AER.

| Model Name | ALL | NAT | GHG | AER | SSP | | | | CTL |
|---|---|---|---|---|---|---|---|---|---|
| | | | | | 1-2.6 | 2-4.5 | 3-7.0 | 5-8.5 | |
| ACCESS-CM2 | 1 | | | | | | | | 12 |
| ACCESS-ESM1-5* | 2 | 2 | 2 | 2 | 2 | 2 | 2 | 2 | 20 |
| AWI-ESM-1-1-LR | | | | | | | | | 2 |
| BCC-CSM2-MR* | 1 | 1 | 1 | 1 | 1 | 1 | 1 | 1 | 14 |
| BCC-ESM1 | | | | | | | | | 10 |
| CAMS-CSM1-0 | 2 | | | | | | | | 12 |
| CanESM5* | 30 | 30 | 30 | 30 | 30 | 30 | 30 | 30 | 24 |
| CanESM5-CanOE | 3 | | | | | | | | - |
| CAS-ESM2-0 | | | | | | | | | 12 |
| CESM2 | 3 | | | | | | | | 28 |
| CESM2-FV2 | | | | | | | | | 12 |
| CESM2-WACCM | 2 | | | | | | | | 12 |
| CESM2-WACCM-FV2 | | | | | | | | | 12 |
| CNRM-CM6-1* | 6 | 6 | 6 | 6 | 6 | 6 | 6 | 6 | - |
| CNRM-CM6-1-HR | 1 | | | | | | | | 6 |
| CNRM-ESM2-1 | 1 | | | | | | | | 12 |
| E3SM-1-0 | | | | | | | | | 12 |
| E3SM-1-1 | | | | | | | | | 4 |
| E3SM-1-1-ECA | | | | | | | | | 4 |
| EC-Earth3 | | | | | | | | | 12 |
| EC-Earth3-LR | | | | | | | | | 4 |
| EC-Earth3-Veg | | | | | | | | | 12 |
| FIO-ESM-2-0 | 3 | | | | | | | | 14 |
| GFDL-CM4 | 1 | | | | | | | | 12 |
| GFDL-ESM4* | 1 | 1 | 1 | 1 | 1 | 1 | 1 | 1 | 12 |
| HadGEM3-GC31-LL* | 4 | 4 | 4 | 4 | 1 | 4 | | 4 | 12 |
| HadGEM3-GC31-MM | | | | | | | | | 8 |
| INM-CM4-8 | 1 | | | | | | | | 12 |

| | | | | | | | | | |
|---|---|---|---|---|---|---|---|---|---|
| INM-CM5-0 | 1 | | | | | | | | 28 |
| IPSL-CM6A-LR* | 7 | 7 | 7 | 7 | 5 | 7 | 7 | 5 | 28 |
| MIROC6* | 3 | 3 | 3 | 3 | 3 | 3 | 3 | 3 | 18 |
| MIROC-ES2L | 1 | | | | | | | | 12 |
| MPI-ESM-1-2-HAM | | | | | | | | | 18 |
| MPI-ESM1-2-HR | 2 | | | | | | | | 12 |
| MPI-ESM1-2-LR | 10 | | | | | | | | 24 |
| MRI-ESM2-0* | 3 | 3 | 3 | 3 | 1 | 1 | 3 | 1 | - |
| NorCPM1 | | | | | | | | | 12 |
| NorESM2-LM* | 3 | 3 | 3 | 3 | 1 | 3 | 1 | 1 | 12 |
| NorESM2-MM | | | | | | | | | 12 |
| SAM0-UNICON | | | | | | | | | 16 |
| UKESM1-0-LL | 5 | | | | | | | | 18 |
| Count (Model/ensemble or chunks) | 25 /97 10*/60* | 10/60 | 10/60 | 10 /60 | 10/51 | 10/58 | 9/54 | 10/54 | 38/516 |
